# Antimicrobial activity and safety evaluation of peptides isolated from the hemoglobin of chickens

**DOI:** 10.1186/s12866-016-0904-3

**Published:** 2016-12-05

**Authors:** Fengjiao Hu, Qiaoxing Wu, Shuang Song, Ruiping She, Yue Zhao, Yifei Yang, Meikun Zhang, Fang Du, Majid Hussain Soomro, Ruihan Shi

**Affiliations:** 1 Department of Veterinary Pathology and Public Health, Key Laboratory of Zoonosis of Ministry of Agriculture College of Veterinary Medicine, China Agricultural University, Beijing, 100193 China; 2 Beijing Huadu Broiler Corporations, Beijing, 102211 China

**Keywords:** Antimicrobial peptides, Hemoglobin, Hydrolysis, Antimicrobial activity, Bactericidal activity

## Abstract

**Background:**

Hemoglobin is a rich source of biological peptides. As a byproduct and even wastewater of poultry-slaughtering facilities, chicken blood is one of the most abundant source of hemoglobin.

**Results:**

In this study, the chicken hemoglobin antimicrobial peptides (CHAP) were isolated and the antimicrobial and bactericidal activities were tested by the agarose diffusion assay, minimum inhibitory concentration (MIC) analysis, minimal bactericidal concentration (MBC) analysis, and time-dependent inhibitory and bactericidal assays. The results demonstrated that CHAP had potent and rapid antimicrobial activity against 19 bacterial strains, including 9 multidrug-resistant bacterial strains. Bacterial biofilm and NaCl permeability assays, transmission electron microscopy (TEM) and scanning electron microscopy (SEM) were further performed to detect the mechanism of its antimicrobial effect. Additionally, CHAP showed low hemolytic activity, embryo toxicity, and high stability in different temperatures and animal plasma.

**Conclusion:**

CHAP may have great potential for expanding production and development value in animal medication, the breeding industry and environment protection.

**Electronic supplementary material:**

The online version of this article (doi:10.1186/s12866-016-0904-3) contains supplementary material, which is available to authorized users.

## Background

Due to the widespread use and even abuse of conventional antibiotics, antibiotic resistance is rampant all over the world, which limits the lifespan of commercial antibiotics and results in the urgent demand of new platforms for efficient antibiotic discovery [1, 2].

As an essential part of innate immunity, antimicrobial peptides (AMPs) have been receiving increasing attention because of their unique antimicrobial mechanism against both Gram-positive and Gram-negative bacteria, and even including some multidrug-resistant strains over recent decades [3, 4]. Antimicrobial peptides are ubiquitous in all living organisms. More than 5000 AMPs (http://www.camp.bicnirrh.res.in/index.php) have been identified and 2593 peptides have been derived naturally (http://aps.unmc.edu/AP/main.php) since the discovery of the lysozyme by Alexander Fleming in 1922 [5]. Although substantial AMPs have been discovered over the past decades, only a small part of them have been used because of high costs and potential cytotoxicity [1, 6]. Hence, finding efficient, nontoxic and low-cost AMPs is urgent in promoting AMPs’ practical applications.

The whole blood is a mixture of cells (erythrocytes, leucocytes and platelets) and plasma (colloids and crystalloids), which delivers nourishment and oxygen to and removes waste products from all parts of the body [7]. Components in blood, such as platelet concentrates [8], defensins [3], leukocyte extracts [9], also play important roles in antimicrobial host defense. Hemoglobin is the main component of the erythrocyte [10]. Aside from the basic function of transporting oxygen, hemoglobin has been found as a source of various biological peptides [11–13]. Many AMPs called hemocidins have been isolated from hemoglobin cleavage *in vivo* [14] or from hemoglobin hydrolysis by chemical reagents, physical methods, or enzymes *in vitro* [15, 16]. To date, the hemocidins derived from human beings [17], bovines [12], rabbits [18], swine [15], crocodiles [19], fish [20], and shellfish [21] have been reported and most of them are made up of 2 to 60 residues, characterized by a common random coil structural and broad-spectrum antimicrobial activity [22–24]. As a byproduct and even one of the major dissolved pollutants in slaughter house wastewater [25], appropriate treatment of chicken blood is of great benefit to both environmental protection and economic development. However, hemocidins from poultry have not been documented yet. In this study, the hemocidins from chickens were isolated and their antimicrobial and bactericidal activities were further detected.

## Methods

### Materials and chemicals

All common chemical reagents and biological products were of analytical grade from commercial sources. Papain (2000 IU g^−1^) was purchased from Sigma Chemical Co. (St. Louis, Mo, America).

### Preparation of CHAP

The chicken hemoglobin antimicrobial peptides (CHAP) were prepared as modified method described before [26]. In brief, fresh chicken blood (Beijing Huadu Broiler Corporations, Beijing, China) was collected with heparin and then centrifuged with 2,000 × *g* at 4 °C for 10 min. The upper liquid and white cells were removed and washed with sterilized saline. The procedures described above were repeated 3 times. The cells were frozen, thawed, stirred and homogenized in deionized water (pH 7.0) with papain (1:1,000 *w/v*) proteolysis at 70 °C for 8 h. The digested suspensions were added with ice-cold aqueous 5% acetic acid solution (1:1 *v/v*) and extracted overnight at 4 °C. After being centrifuged at 8,000 × *g* for 30 min at 4 °C, the suspensions were collected as crude extracts, and the protein concentration was detected by NanoDrop 2000 UV–vis Spectrophotometer (Thermo Fisher Scientific, Massachusetts, America). The pH of the extracts was adjusted to 6.0 with sodium hydroxide. The crude extracts were loaded onto 10 × 300 mm Sephadex G-100 column and eluted by 0.2 mol L^−1^ sodium acetate buffer (pH 6.0) with the speed of 12.0 mL cm^−2^ h ^-1^_._ Each elution was analyzed by agarose diffusion assay [27] with *Escherichia coli* ATCC 25922 as the indicator organism. The fractions with potent antibacterial activity were collected and detected with Tricine SDS-PAGE [28] and then subjected to mass spectrometry (Beijing Protein Innovation Co., Ltd., Beijing, China).

### Bacterial strains and growth conditions

*Staphylococcus aureus* ATCC25923, *Staphylococcus aureus* ATCC 29213, *Staphylococcus albus* ATCC01331, *Escherichia coli* ATCC 25922, *Escherichia coli* O78, *Escherichia coli* C83922, *Escherichia coli* C83901, *Pseudomonas aeruginosa* ATCC27853, *Pasteurellae gallinarum* C48-3 were purchased from the China Veterinary Culture Collection Center (CVCC). *Aeromonas hydrophila*, *Bacillus cereus* and *Escherichia coli* were clinically isolated from crucian carps, pigeon and equines respectively by Laboratory of Veterinary Pathology and Public Health of the College of Veterinary Medicine, China Agricultural University. *Staphylococcus aureus* MR-L22, MR-QD-CD10, *Enterococcus faecalis* 53A, 52A, 37 N and *Pseudomonas aeruginosa* M140 and *Escherichia coli* T50 were all multi-resistance strains of clinics, and obtained from Beijing Key Laboratory of Detection Technology for Animal Food safety of the College of Veterinary Medicine. All the above Gram-negative strains were grown in Luria-Bertani (LB) agar and the Gram-positive bacteria were grown in brain heart infusion (BHI) agar.

### Determination of antimicrobial and bactericidal activities

#### Agarose diffusion assay

The primary antibacterial activities of CHAP elution (100 μg mL^−1^) were detected by modified agarose diffusion assay as described before [27]. Briefly, the single colony of each bacterial strain was grown in trypticase soy broth (TSB, 30 g L^−1^) overnight at 37 °C under aerobic conditions. 2 × 10^8^ CFU mL^−1^ bacteria culture of each strain was added to warm (50–55 °C) sterile agarose [1% agarose (low EEO, Sigma, St. Louis, MO), 0.03% nutrient broth, and 10 mM PBS buffer, pH 7.4] (1:100 *v/v*). 10 μL samples were added to 3 mm wells punched by agar punch (BioRad Laboratories, Hercules, Canda). 0.2 mol L^−1^ sodium acetate (solvent) and 20000 IU penicillin-streptomycin solution of the same volume were added as negative and positive control, respectively. After being incubated overnight at 37 °C, the diameter of the each clean zone of growth inhibition was measured as the antibacterial activity of CHAP against different strains.

#### Minimum inhibitory concentration (MIC) analysis

A micro dilution assay was employed to determine MIC according to the broth micro dilution guideline of Clinical and Laboratory Standards Institute (CLSI) [29]. Briefly, 50 μL of twofold serial dilutions of CHAP (25 to 0.20 μg mL^−1^) was placed into wells of sterile 96-well cell culture plates. The 50 μL of bacterial suspensions (1 × 10^5^ CFU mL^−1^) were added to the peptides. The wells were added with 50 μL of Mueller- Hinto (MH) broth and 50 μL of bacterial culture was treated as positive and negative control, respectively. After 24 h incubation, the MICs were determined at 492 nm by spectrophotometer (Thermo Multiskan MK3, Thermo Fisher Scientific, Massachusetts, America).

#### Time-dependent inhibitory assay

A 500 μL aliquot of CHAP with 2 × MIC of the bacterium was added respectively to 500 μL bacterial suspensions (1 × 10^5^ CFU mL^−1^) in the sterilized 1.5 mL tubes as the treated groups. Bacteria treated with 500 μL solvent (0.2 mol L^−1^ sodium acetate) were set as the control groups. After being incubated for 30 min, 100 μL aliquot of the suspensions were pipetted into to a sterilized 1.5 mL tube. After centrifugation at 1,000 × *g* for 5 min, the supernatant was removed, and the pellet was resuspended in 100 μL MHB medium. Tenfold serially diluted suspension was placed on agar plates and incubated at 37 °C until viable colonies could be seen and the numbers of colony-forming units (CFU) were counted. The inhibitory rate of each bacterium was calculated according to the following formula: the inhibitory rate = [(colonies of the treated group - colonies of the treated group)/colonies of the control group] × 100%.

In order to further detect the process and speed of the antimicrobial activity of CHAP, the time growth curves and inhibitory rates of *Escherichia coli* ATCC 25922, *Staphylococcus aureus* ATCC29213, *Staphylococcus aureus* MR-L22, *Enterococcus faecalis* 52A, *Pseudomonas aeruginoda* M140 and *Escherichia coli* T50 were achieved after the treated suspensions were incubated for 0, 5, 10, 30, 90 min respectively.

#### Minimal bactericidal concentration (MBC) analysis

The MBC values were determined in 96-well plates, which was similar to the method of MIC. MBC values were further confirmed by plating 100 μL samples of each well with no visible turbidity onto the MHB medium. The least concentration showing no visible growth on the plates was considered as the MBC value.

#### Time-dependent bactericidal assay

The time depending bactericidal curves of *Escherichia coli* ATCC 25922 were determined as the time-dependent inhibitory assay mentioned above by adding CHAP with concentration of its MBC value to the bacterial cultures grown to early and late exponential phase as the reference [2].

In order to detect the bacteriolysis against bacteria in stationary phase, 10 ml of bacterial culture (2 × 10 ^9^ CFU mL^−1^) was treated with 10 × MIC of CHAP. The culture treated with solvent (0.2 mol L^−1^ sodium acetate) was set as the control group. After 24 h incubation, 2 ml of each culture was added to a glass tube and was photographed [2].

#### Bacterial biofilms assay

Crystal violet staining method was applied to detect the effect of CHAP on the biofilm formation [30]. Briefly, *Staphylococcus aureus* ATCC29213 were cultured in TSB overnight. 100 μL bacterial suspensions (1 × 10^6^ CFU mL^−1^) with 2 × MIC, 1 × MIC, 1/2 × MIC, 1/4 × MIC, 1/8 × MIC, 1/16 × MIC of CHAP were added to 96-well plates and the bacterial suspensions with no CHAP and the sterilized TSB were treated as control groups. After static culture at 37 °C for 24 h or shake culture (50 rmp) at 37 °C for 72 h, the contents were aspirated and the wells were washed by 200 μL PBS for three times, methanol fixed for 1 h and stained with 200 μL crystal violet (5 g L^−1^) for 30 min. The wells were washed by running water and air dried. The plates were determined at 600 nm by spectrophotometer.

#### NaCl permeability assay

The effect of CHAP on the NaCl permeability of bacteria was detected as modified protocol as follows [31]. 100 μL of bacterial suspensions (1 × 10^6^ CFU mL^−1^) with 1/2 × MIC of CHAP were added to 96-well plates and the bacterial suspensions with no CHAP as control. 100 μL of NaCl solutions with different concentrations (80, 100, 120, 140, 160, 180, 200 g L^−1^) were added into each wells and incubated at 37 °C for 12 h. The bacterial concentration of each well was determined by measuring the optical density at 600 nm (OD_600_).

#### Electron microscopy observations

Both transmission electron microscopy (TEM) and scanning electron microscopy (SEM) were conducted as previously described [32–34]. Briefly, *Escherichia coli* ATCC 25922 and *Staphylococcus aureus* ATCC29213 were cultured overnight, 10^7^ CFU ml^−1^ bacteria were incubated with 1 × MIC of CHAP or diluents of the same volume at 37 °C for 30 min. All the samples were fixed and proceeded for the TEM and SEM respectively.

### Hemolytic assay and embryotoxicity assay

The hemolytic activity was evaluated as previously described [35]. 4% (vol/vol) fresh chicken erythrocyte suspensions were added to a 96-well plate and incubated with CHAP at 360, 180, 90, 45, 22.5, 11.25 μg mL^−1^ individually at 37 °C for 1 h. Wells treated with PBS and 0.1% Triton X-100 of the same volume were taken as 0 and 100% hemolysis. The wells were determined by measuring the optical density at 492 nm (OD_492_).

The embryotoxicity of CHAP was detected as the following measures. The 10-days-old-chicken embryos were randomly divided into 5 groups, each of 10 eggs, 0.2 mL of CHAP of 1 × MIC, 2 × MIC, 4 × MIC, 6 × MIC dose against *Escherichia coli* ATCC25922 were injected into the chorio-allantoic cavity, and embryos treated with the same volume of solvent (0.2 mol L^−1^ sodium acetate) were used as controls. The eggs were put in a hatching machine and hatchability and weight of the eggs were observed regularly until hatching.

### Stability in different temperatures and in 50% plasma

CHAP (100 μg mL^−1^) was treated with different temperatures varying from 30 °C, 40 °C, 50 °C, 60 °C, 70 °C, 80 °C, 90 °C, 100 °C, 121 °C for 30 min. The antimicrobial activities of these treated aliquots were determined with agarose diffusion assay and were compared with CHAP stored in 4 °C.

The stability of CHAP in 50% plasma was evaluated as previously described [29] with some modifications. Briefly, the plasma of chicken and rabbit was determined with no antimicrobial activity before the test. Then 640 μg ml^−1^ CHAP was diluted 1:1 in fresh chicken and rabbit plasma and pre-incubated at 37 °C for 0, 3, and 6 h respectively. After incubation, the antimicrobial activity of each sample was determined by agarose diffusion assay. The effect of CHAP diluted by its solvent was regarded as the 100%, and the effect of the treated samples was demonstrated as percentages.

### Statistical Analysis

Experiments were conducted with biological replicates and experimental data were expressed as mean ± standard deviation of at least three determinations and analyzed by one-way ANOVA using SPSS 20.0 (SPSS Inc., Cary, NC, USA). Differences were considered to be statistically significant at *P* < 0.05 or *P* < 0.01.

## Results

### Preparation of CHAP

**Fig. 1:**
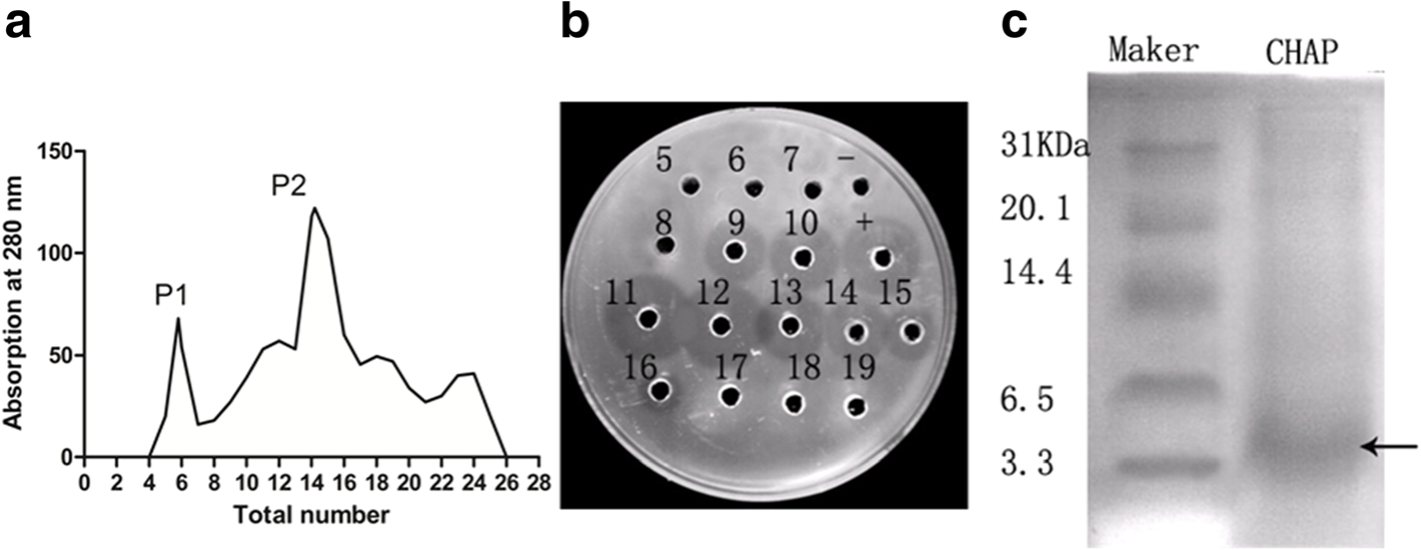
Preparation of CHAP. **a** Sephadex G-100 gelatin separation of the extract from chicken blood. **b** Antibacterial activity detection of elution of Sephadex G-100 gelatin by agarose diffusion assay against *Escherichia coli* ATCC25922. **c** Tricine–SDS-PAGE of the interested elution and the band was around 3.3 KDa (*arrow*)

The crude extracts of CHAP were light yellow and the protein concentration was adjusted to 5 mg ml^−1^ before loading on the Sephadex G-100 column. There were two main peaks after the elution of Sephadex G-100 gelatin (Fig. 1a) and the tubes from 9 to 16 in the left half of the second peak showed potent antimicrobial activity (Fig. 1b). Detected by Tricine SDS –PAGE, the collected CHAP showed band around 3.3 KDa (Fig. 1c). This band was further analyzed and peptides of gallus hemoglobin subunit alpha were confirmed by mass spectrum (see Additional file 1).

### Determination of antimicrobial and bactericidal activity

#### Antimicrobial effect of CHAP

The results of antibacterial activity of CHAP detected via agarose diffusion assay, MIC assay and inhibitory rate assay are shown in the columns 2–4 in Table 1.These results demonstrated that CHAP performed potent antimicrobial activities against both Gram-negative bacteria and Gram-positive bacteria, including 9 multidrug-resistant strains.

**Table 1:** Antibacterial activity and bactericidal activity of Chicken hemoglobin fragment peptides

Strains	D (mm)	MIC (μg mL^−1^)	IR (%)	MBC (μg mL^−1^)
Gram-negative bacteria				
*Escherichia coli* ATCC 25922	24.5	6.25	83.00	80
*Escherichia coli* C83901	18	12.5	66.53	80
*Escherichia coli* C83922,	20	6.25	55.24	80
*Escherichia coli* O78	18	6.25	47.83	80
*Aeromonas hydrophila*(crucian carp)	13	3.13	52.05	>160
*Pseudomonas aeruginosa* ATCC27853	15	3.13	52.94	80
*Pasteurellae gallinarum* C48-3	12	3.13	63.63	80
**MR-** ***Escherichia coli*** **(equine)**	33.5	6.25	62.73	80
**MR-** ***Pseudomonas aeruginosa*** **M140**	19	1.56	85.56	5
**MR-** ***Escherichia coli*** **T50(swine)**	11	6.25	89.92	80
Gram-positive bacteria				
*Staphylococcus aureus* ATCC25923	27	3.13	50.00	>160
*Staphylococcus aureus* ATCC 29213	21	3.13	94.26	5
*Staphylococcus albus* ATCC01331	14.5	1.56	73.36	40
**MR-** ***Bacillus cereus*** **(pigeon)**	13.5	3.13	73.36	20
**MR-** ***Staphylococcus aureus*** **L22(swine)**	15.5	6.25	88.6	>160
**MR** ***-Staphylococcus aureus*** **QD-CD10 (swine)**	15.5	6.25	56.93	>160
***MR-Enterococcus faecalis*** **53A(pet)**	22	1.56	91.10	>160
***MR-Enterococcus faecalis*** **52A(pet)**	13.5	6.25	35.00	>160
***MR-Enterococcus faecalis*** **37 N(pet)**	29	3.13	55.00	>160

*MR* multidrug resistance (in bold), *D* diameter of inhibition zone, *MIC* minimum inhibitory concentration, *IR* inhibitory rate in 30 min, *MBC* minimal bactericidal concentration

**Fig. 2:**
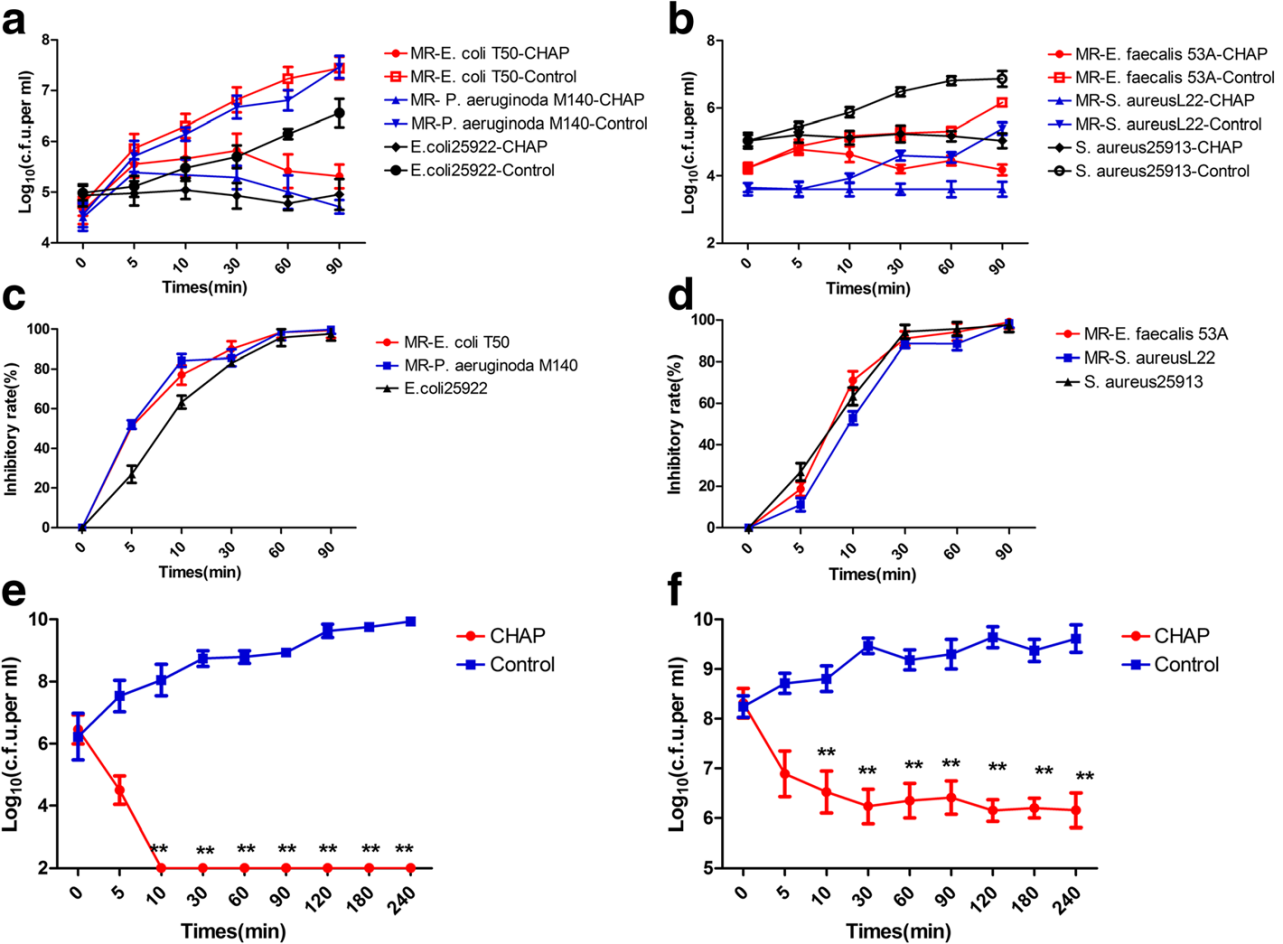
**a**-**b** Time-dependent inhibitory curves of bacteria treated with CHAP and the bacteria treated with the solvent as control. **a** Gram-negative bacteria. **b** Gram-positive bacteria. **c**-**d** Time-dependent growth inhibitory rate curves of bacteria treated with CHAP and the bacteria treated with the solvent as control. **c** Gram-positive bacteria. **d** Gram-positive bacteria. **e**-**f** Time dependent bactericidal curves of *Escherichia coli* ATCC 25922 treated with CHAP and the bacteria treated with the solvent as control. **e** At early exponential phase. **f** At late exponential phase

The time-dependent growth inhibitory activities of CHAP are shown in Fig. 2a-d. The results showed that CHAP not only significantly inhibited the growth of standard strains (*P* < 0.05), but also effectively inhibited the multi-resistant ones (*P* < 0.05) in 10 min (Fig. 2a and b). The inhibitory rates of all six strains reached 100% in 90 min (Fig. 2c and d). Although there were different growth inhibitory curves, CHAP showed more than 50% inhibitory rate against all six strains in 10 min.

#### Bactericidal effect of CHAP

**Fig. 3:**
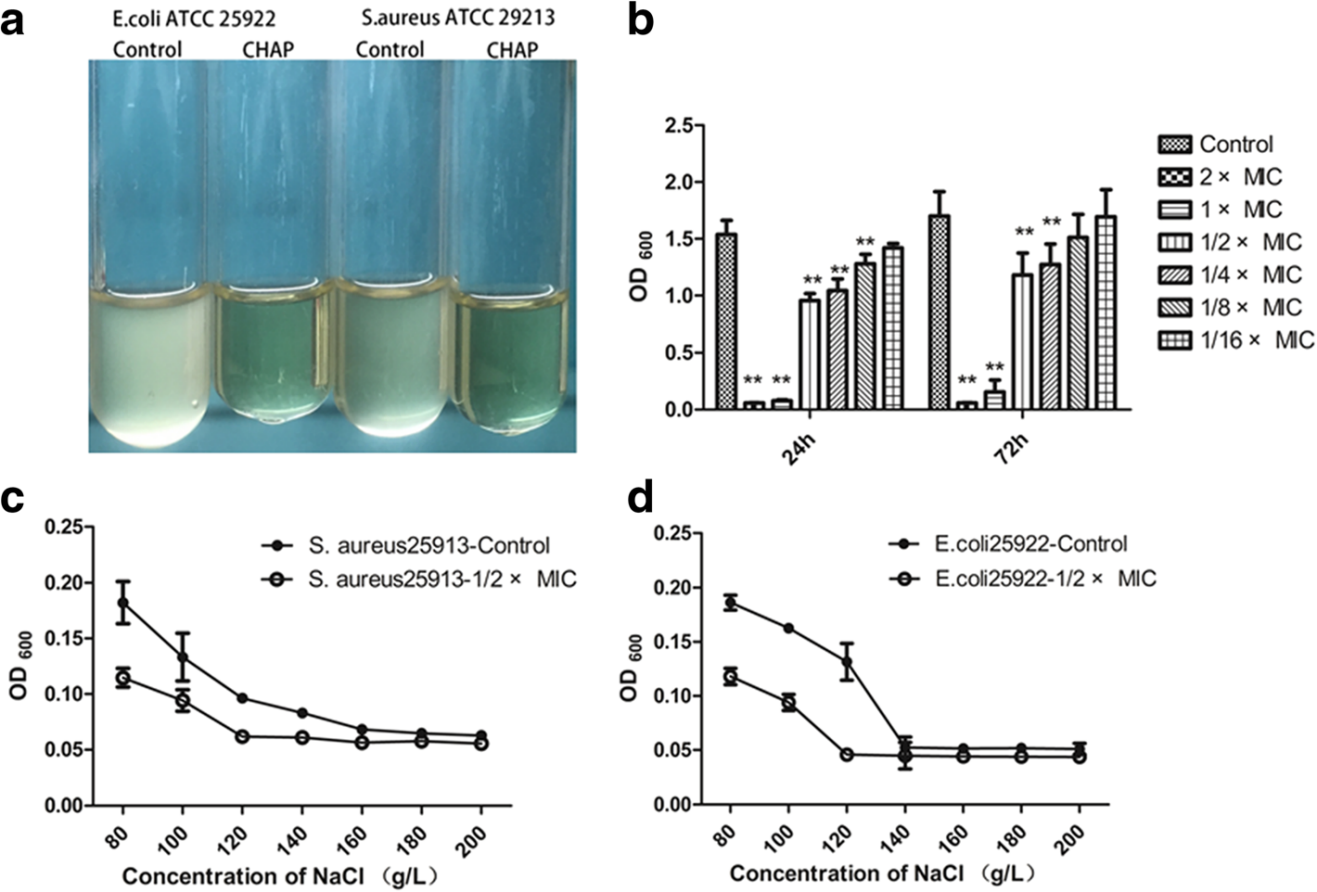
**a** Bacteriolysis analysis. Bacteria in stationary phase treated with CHAP resulted in lysis with the solvent as control. **b** The inhibitory effect of CHAP on bacterial biofilm for 24 h and 72 h. **c**-**d** The effect of CHAP on NaCl permeability of *Escherichia coli* ATCC 25922 and *Staphylococcus aureus* ATCC29213

The values of MBC are shown in column 5 of Table 1. By analyzing the values, most bacteria were killed by CHAP at concentrations ranging from 5 μg mL^−1^ to 80 μg mL^−1^. However, 7 strains showed no obvious bactericidal effect with the maximum concentration of 160 μg mL^−1^. The time-dependent bactericidal curves in Fig. 2e and f further revealed that the significant bactericidal effect of CHAP on both the bacteria grown to early and late exponential phases from 10–240 min (*P* < 0.01). Especially the early exponential phase bacteria, they were killed completely in only 10 min. The bacteria in stationary phase resulted in lysis after being treated with CHAP for 24 h (Fig. 3a).

#### Bacterial biofilms assay

The formation of biofilms of *Staphylococcus aureus* ATCC29213 was decreased with the increase of the concentration of CHAP (Fig. 3b). The 2 × MIC, 1 × MIC, 1/2 × MIC, 1/4 × MIC, 1/8 × MIC of CHAP could inhibited the formation of biofilms (*P* < 0.01) in 24 h and 2 × MIC, 1 × MIC, 1/2 × MIC, 1/4 × MIC of CHAP could significantly decrease the formation of biofilms (*P* < 0.01) in 72 h. Remarkably, in the 2 × MIC and 1 × MIC groups, there were almost no biofilm formation.

#### NaCl permeability assay

As shown in Fig. 3c and d, the values of OD_600_ of *Escherichia coli* ATCC 25922 and *Staphylococcus aureus* ATCC29213 cultures decreased with the increased concentration of NaCl solution and reached to their minimum values at concentration above 160 g L^−1^ and above 140 g L^−1^, respectively. By adding CHAP, the value of OD_600_ in both *Escherichia coli* ATCC 25922 and *Staphylococcus aureus* ATCC29213 groups decreased to the lowest value at concentration of NaCl above 120 g L^−1^.

#### Electron microscopy observations

**Fig. 4:**
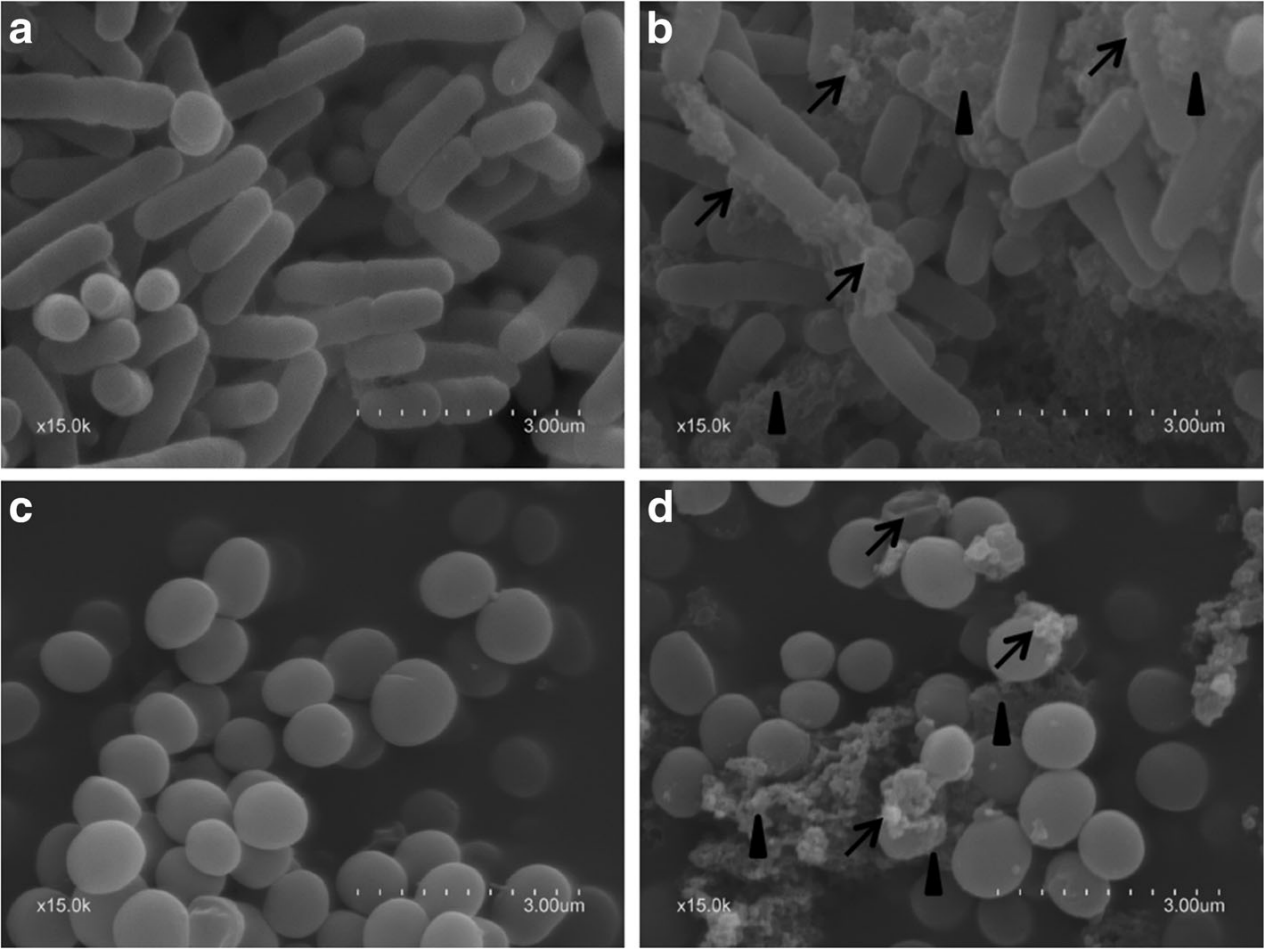
The morphology of *Escherichia coli* ATCC 25922 and *Staphylococcus aureus* ATCC29213 were investigated by scanning electron microscopy. **a**-**b** The control group. **c**-**d** The *Escherichia coli* ATCC 25922 treated with CHAP. Viscous substances were adhering to almost all CHAP treated cells, which got large number of bacteria together (*arrowheads*); Some bacteria showed variable length, rough cell surfaces or globular protrusions on their surfaces, and even appeared to collapse (*arrows*)

**Fig. 5:**
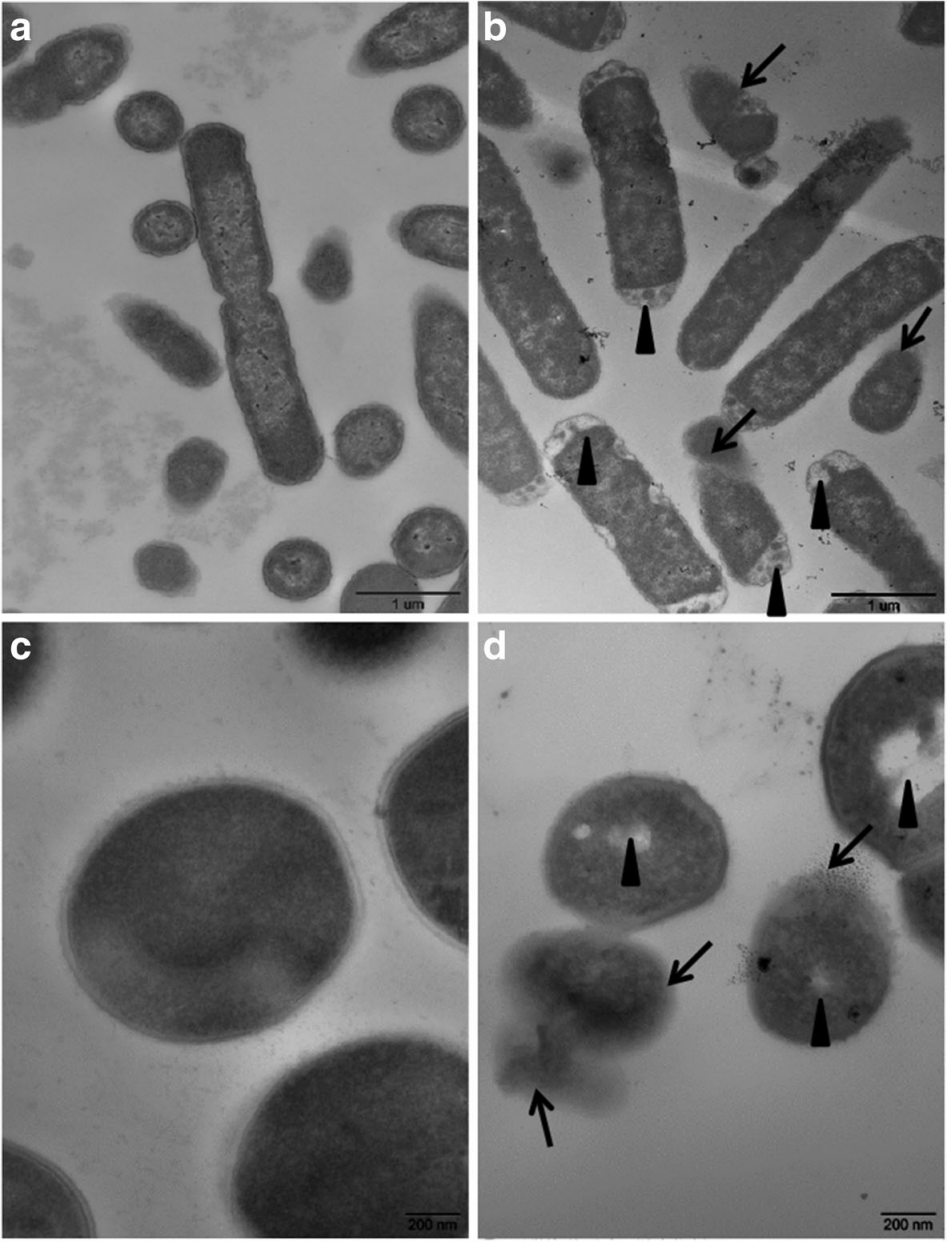
The morphology of *Escherichia coli* ATCC 25922 *Staphylococcus aureus* ATCC29213 were investigated by transmission electron microscopy. **a**-**b** The control group. **c**-**d** The *Escherichia coli* ATCC 25922 treated with CHAP. Most of the bacteria were translucent and pores were evident on walls especially at the two terminals of each cell (*arrowheads*). There was some intracellular substance released from many bacteria (*arrows*).

The morphology of the *Escherichia coli* ATCC 25922 and *Staphylococcus aureus* ATCC29213 investigated by SEM is shown in the Fig. 3. Compared to the smooth, straight and unbroken surface of the control cells (Fig. 4a and c), the strains treated with CHAP for 30 min appeared severely damaged (Fig. 3b and d). The TEM images further demonstrated that the bacterial surfaces were damaged by the effect of CHAP (Fig. 5a and c) compared with the control group (Fig. 4b and d).

### Hemolytic assay and embryotoxicity assay

The hemolysis of CHAP was 38.9% at the concentration of 360 μg mL^−1^ which was more than 50 times higher than the MIC values for all the detected bacteria (see Additional file 2). And the embryotoxicity assay showed that even CHAP of 6 × MIC dose against *Escherichia coli* ATCC25922 did not induce toxicity toward chicken embryos, that is, there was no dead or significant decrease of body weight compared to the control group (*P* > 0.05) (see Additional file 3).

### Stability in different temperatures and in 50% plasma

**Fig. 6:**
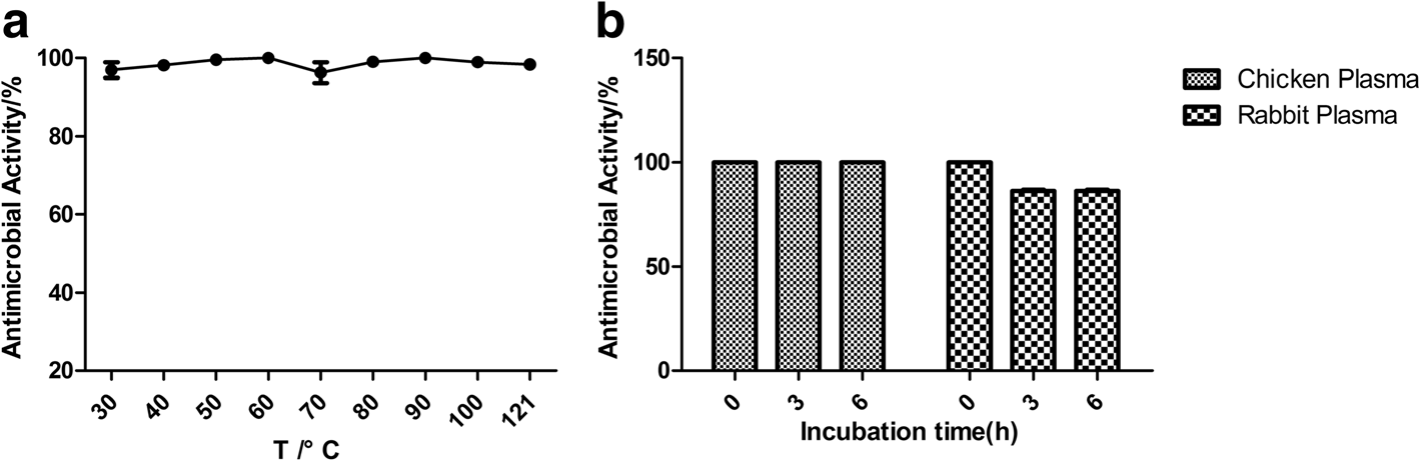
The stability of CHAP. **a** The stability of the CHAP in different temperatures. **b** The stability of the CHAP in chicken and rabbit plasma

The antimicrobial activity of CHAP did not decrease in different temperatures even when it was treated in 121 °C for 30 min compared with CHAP stored in 4 °C (*P* > 0.05) (Fig. 6a). It well proved that CHAP was capable of stability in various temperatures.

Compared with CHAP diluted in the solvent, the antimicrobial activity of CHAP showed no change in the treatment of chicken plasma and a slight but no significant decrease in treatment of rabbit plasma (*P* >0.05) (Fig. 6b), which demonstrated that CHAP was of well stability in the plasma.

## Discussion

Since the first anti-Streptococcus peptide was identified from the cow’s milk [36], the enzyme strategy of isolating AMPs on a large scale has been a feasible method as Bolscher postulated [37]. Our lab isolated the peptides from the hemoglobin of chickens by using a simple and practical way and studied the antimicrobial activities against 19 bacterial strains, including 9 multidrug-resistant bacteria. At the same time, the properties such as hemolytic activity, embryotoxicity and stability in different temperatures and plasma were detected, which laid a foundation for its further employment in agricultural production, public health and medication.

Antibiotics have been helping humans to fight against hazardous infections since Alexander Fleming discovered the first antibiotic, penicillin, in 1928 [38]. However, resistance to most antibiotics was discovered shortly after their applications. For example, penicillin resistance arose in 1946 just one year after its introduction to clinics [1]. The main target of most AMPs is cell membrane and there are several models for explaining the process such as barrel-stave pore model, thoroidal pore model and carpet model [3, 29, 39], and it tends to be difficult for bacteria to totally change this basic structure to resist the effect of AMPs [3]. Given the unique antimicrobial mechanism of AMPs, it was not surprising that most AMPs induced little or no resistance [40, 41]. Although there are reports and doubts about the resistance of some peptides [41, 42], there is no report about the natural ones so far, which means the development of natural AMPs is high in potential.

In our study, both the antimicrobial and bactericide results showed that CHAP was capable of strong and rapid activities against various bacteria and even some multi-resistant strains, implying its wider utility in the prevention and treatment of infectious agents, which was similar with the hemosidins reported before [16, 17]. By specifically analyzing the results above, there was no obvious difference between the antibacterial activity against Gram-negative bacteria and Gram-positive bacteria, even between the standard strains and the multi-resistant strains, suggesting that the target of CHAP is the common component of bacteria such as the cell membrane like most AMPs reported before [29, 43]. The biofilm and the NaCl permeability results showed that CHAP could inhibit the formation of bacterial biofilms and change the permeability of some Gram-negative bacteria and Gram-positive bacteria to some extent. With the confirmation of the mechanism of most AMPs, the EM observations further revealed that CHAP could accumulate copious pathogens nearby and punch through their cell surfaces swiftly [29, 32]. However the specific mechanism needs to be further investigated.

As a double-edged sword, the unique mechanism of targeting cell membranes could also lead to the low selection of some AMPs [3]. Hence, toxicity especially hemolysis and safety problems, are constantly an obstacle to their final applications [1, 40, 44]. According to our study, CHAP demonstrated low hemolysis and embryotoxicity even at rather high concentrations, which further implied that there was relative high selectivity of CHAP between eukaryote cells and prokaryote cells.

Good stability also plays an important role in the application of any biological product. As for the AMPs, the substances such as ions and proteolytic enzymes in the serum may reduce their biological ability to a large extent [29, 45]. In this study, CHAP kept high antimicrobial activity in two kinds of animal serum and different temperatures, suggesting its convenient application, transportation and storage.

## Conclusions

In summary, this study firstly reported a practical method of isolating chicken hemosidins (CHAP) from the byproduct and even the pollutant of chicken-slaughtering industries. CHAP has an attractive antimicrobial and bactericidal ability with low hemolysis, low or none in toxicity and good temperature resistance and high stability in serum, which well accounts for their potential of expanding production and high development value in animal medication, breeding industry and environment protection.

## Additional files

Additional file 1: The mass spectrum result of CHAP. (PDF 18 kb)
Additional file 2: Table S1. Hemolysis of CHAP. (PDF 143 kb)
Additional file 3: Figure S1. Results of the embryotoxicity assay. (PDF 112 kb)

## Abbreviations

AMPs: Antimicrobial peptides
BHI: Brain heart infusion
CFU: Colony-forming units
CHAP: Chicken hemoglobin antimicrobial peptides
CLSI: Clinical and Laboratory Standards Institute
CVCC: China Veterinary Culture Collection Center
LB: Luria-Bertani
MBC: Minimal bactericidal concentration
MH: Mueller- Hinto
MIC: Minimum inhibitory concentration
OD: Optical density
SEM: Scanning electron microscopy
TEM: Transmission electron microscopy
TSB: Trypticase soy broth
