# Supplementary material for: Antimicrobial activity and safety evaluation of peptides isolated from the hemoglobin of chickens
Source: BMC Microbiol. 2016 Dec 5;16:287. doi: 10.1186/s12866-016-0904-3 (PMC5139128; doi:10.1186/s12866-016-0904-3)
Supplement: Additional file 1: — The mass spectrum result of CHAP. (PDF 18 kb) [file 12866_2016_904_MOESM1_ESM.pdf]

Result of Mass Spectrum

From: Mascot Search Results
Match to: **P02001** Score: **731**
Hemoglobin subunit alpha-D OS=Gallus gallus GN=HBAD PE=1 SV=1

Nominal mass (M_r): **15742**; Calculated pI value: **7.01**
NCBI BLAST search of P02001 against nr
Unformatted sequence string for pasting into other applications

Fixed modifications: Carbamidomethyl (C)
Variable modifications: Gln->pyro-Glu (N-term Q),Oxidation (M)
Cleavage by Trypsin: cuts C-term side of KR unless next residue is P
Sequence Coverage: **83%**

Matched peptides shown in **Bold Red**

```
  1 MLTAEDKKLI QQAWEKAASH QEEFGAEALT RMFTTYPQTK TYFPHFDLSP
 51 GSDQVRGHGK KVLGALGNAV KNVDNLSQAM AELSNLHAYN LRVDPVNFKL
101 LSQCIQVVLA VHMGKDYTPE VHAAFDKFLS AVSAVLAEKY R
```
