# Supplementary material for: Antimicrobial activity and safety evaluation of peptides isolated from the hemoglobin of chickens
Source: BMC Microbiol. 2016 Dec 5;16:287. doi: 10.1186/s12866-016-0904-3 (PMC5139128; doi:10.1186/s12866-016-0904-3)
Supplement: Additional file 2: Table S1. — Hemolysis of CHAP. (PDF 143 kb) [file 12866_2016_904_MOESM2_ESM.pdf]

Table S 1 Hemolysis of CHAP

The Concentration of CHAP(μg mL^{-1})	Absorbance at 492nm	Hemolysis values(%)
360.00	0.134483±0.003434	38.94
180.00	0.128289±0.011835	35.11
90.00	0.100211±0.004465	17.75
45.00	0.079056±0.013379	4.67
22.50	0.078689±0.008958	4.45
11.25	0.074878±0.009262	2.09
6.13	0.073156±0.005967	1.03
3.06	0.073689±0.008074	1.36
Positive	0.23325±0.047981	100
Negative	0.071405±0.010793	0

Note: The Hemolysis values (%) were calculated as mentioned in the method that is Hemolysis values=(A-N)×100/(P-N) and A=Absorbance of different concentrations of GHAP, N=Absorbance of negative group, P=Absorbance of positive group
