# Supplementary material for: Antimicrobial activity and safety evaluation of peptides isolated from the hemoglobin of chickens
Source: BMC Microbiol. 2016 Dec 5;16:287. doi: 10.1186/s12866-016-0904-3 (PMC5139128; doi:10.1186/s12866-016-0904-3)
Supplement: Additional file 3: Figure S1. — Results of the embryotoxicity assay. (PDF 112 kb) [file 12866_2016_904_MOESM3_ESM.pdf]

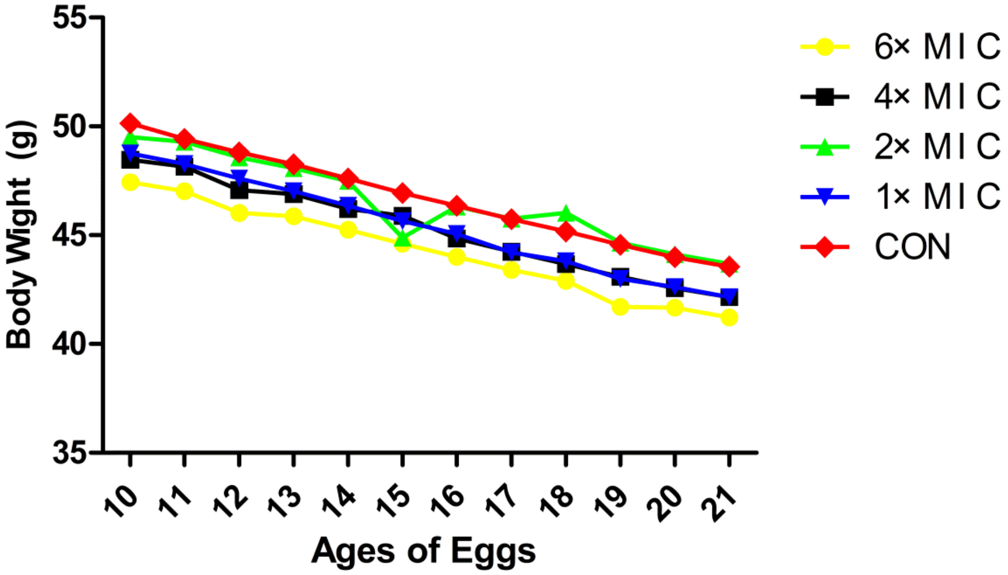

Fig. S1 Results of embryotoxicity assay. There was no death in all the groups and no significant difference in the body weight of CHAP treated groups compared with the control group ($P > 0.05$).
